# Time-Dependent Effects of PFAS and Their Mixtures on Ovarian Epithelial Cell Proliferation and Chemotherapeutic Response

**DOI:** 10.3390/ijms27146301

**Published:** 2026-07-15

**Authors:** Robinson Ajana, Dominik Rachoń, Grażyna Gałęzowska

**Affiliations:** 1 Department of Clinical and Experimental Endocrinology, Faculty of Health Sciences, Medical University of Gdańsk, 80-211 Gdańsk, Poland; robinson.ajana@gumed.edu.pl (R.A.); dominik.rachon@gumed.edu (D.R.); 2 Division of Bioenergetics and Physiology of Exercise, Faculty of Health Sciences, Medical University of Gdańsk, 80-211 Gdańsk, Poland; 3 Department of Environmental Engineering Technology, Faculty of Civil and Environmental Engineering, Gdańsk University of Technology, 80-233 Gdańsk, Poland

**Keywords:** PFOA, PFOS, cell proliferation, cabazitaxel, ovarian epithelial cells

## Abstract

Per- and polyfluoroalkyl substances (PFAS) are an emerging group of anthropogenic environmental pollutants frequently detected in human and animal serum. Although several studies have reported adverse health effects associated with exposure to single PFAS, limited information is available concerning the biological and toxicological impact of PFAS mixtures and their potential interactions with chemotherapeutic agents. This study investigated the effects of acute and chronic exposure to PFAS, including perfluorooctanoic acid (PFOA, 3 nmol/L), perfluorooctane sulfonic acid (PFOS, 5 nmol/L), and their binary mixture containing 3 nmol/L of PFOA plus 5 nmol/L of PFOS, using an in vitro model, mouse ovarian surface epithelial cells (MOSEC), and possible interactions with antineoplastic agents. Results showed increased cell proliferation following acute PFOS exposure; this effect was enhanced in the PFOA-PFOS mixture. In contrast, PFOA exposure showed reduced cell proliferation. Acute exposure to PFOA, PFOS, and their mixture was associated with reduced sensitivity to cabazitaxel and docetaxel, while chronically exposed cells demonstrated increased sensitivity. These findings suggest that PFOA, PFOS, and their mixtures present in human serum and tissues may influence cellular proliferation and alter therapeutic response. These findings further highlight the importance of mixture effects and the exposure duration effect in the toxicological evaluation of PFAS toxicity.

## 1. Introduction

Per- and polyfluoroalkyl substances (PFAS) are a complex group of man-made persistent organofluoride compounds. The unique carbon–fluorine bond confers chemical and thermal stability, making PFAS useful in various fields, such as cosmetics, agriculture, textiles, food packaging, and household utensils [1], thereby making them ubiquitous environmental pollutants.

PFAS are released into the environment through physical abrasion and degradation of fluorinated compounds [2], resulting in human exposure via various routes, such as oral [3,4,5], transdermal [6], and inhalation [7]. Due to their resistance to degradation [8,9], PFAS persist in the environment [10] and in human body fluids and tissues.

PFAS accumulation in tissues such as the brain, liver, kidney, bone, and lung [11] has been linked to an increased risk of thyroid, kidney, testicular, and breast neoplasms [12,13,14,15]. PFAS accumulation in follicular fluid has been associated with delayed menarche [16,17,18], menstrual irregularities [19], and increased risk of premature ovarian failure [20,21].

Current knowledge on the reproductive toxicity of PFAS has been outlined in our previous review article [22].

PFAS exposure has shown not only an impact on the number of ovarian follicles in juvenile and adult animal models [23,24] but also induces granulosa cell proliferation and alters the cell cycle in human cell lines [24,25,26,27,28]. PFAS exposure may have been associated with ovarian carcinogenesis, platinum-resistant ovarian cancer [29,30,31], and increased prostate cancer cell proliferation in an in vitro study [32].

Recent studies have reported that PFAS exposure may induce oxidative-stress-mediated DNA damage [33], induce drug resistance in colorectal cancer [34], and induce chemotherapy resistance in ovarian cancer [35]. Our previous review article and meta-analysis demonstrated that, irrespective of PFAS type, exposure is associated with different adverse health outcomes [22], especially legacy PFAS like perfluorooctanoic acid (PFOA) and perfluorooctane sulfonic acid (PFOS).

PFOA/PFOS are a perfluoro class of PFAS with an eight-carbon chain attached to a carboxylic and sulfonic functional group, respectively [36,37]. They are among the most extensively studied PFAS groups. Both compounds bioaccumulate in tissues and are associated with reproductive toxicity, increasing the risk of cancer [22]. However, numerous studies have examined the toxicological effects of individual PFAS. Limited information is available regarding the comparative and cumulative effects of environmentally relevant low-dose PFOA and PFOS, the most frequently detected legacy PFAS in human serum [38]. The current toxicological framework has not clearly demonstrated how exposure duration influences ovarian cell proliferation and chemotherapeutic response following combined and individual PFAS exposure at a human-serum-inspired low concentration.

Given the persistent and bioaccumulating nature of PFOA/PFOS, comparing the acute and chronic effects may provide insight into both immediate and long-term cellular responses.

This study aimed to address the existing knowledge gap by investigating the potential time-dependent effects of PFOA, PFOS, and their mixture on cell proliferation at environmentally relevant concentrations derived from reported human serum exposure ranges, as well as their potential interaction with anti-microtubule chemotherapeutic agents docetaxel and cabazitaxel, using mouse ovarian surface epithelial cells (MOSECs).

## 2. Results

**Figure 1:**
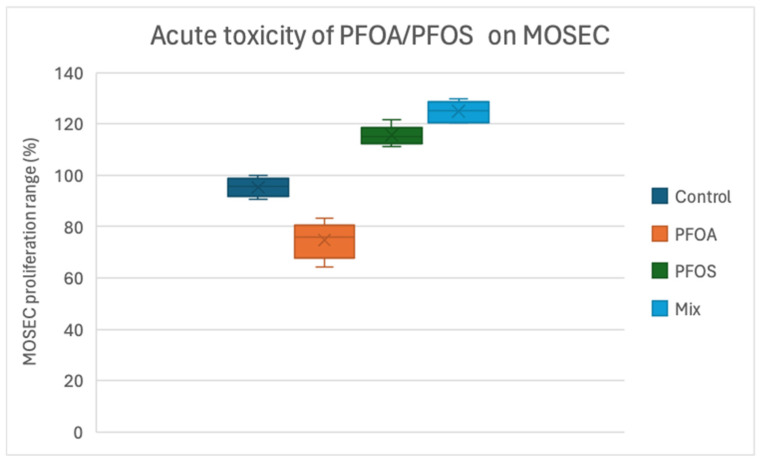
Acute toxicity of PFOA/PFOS on MOSEC: The cell lines were exposed to 3 nmol/L of PFOA, 5 nmol/L of PFOS, and their mixture (3 nmol/L of PFOA + 5 nmol/L of PFOS). Cell proliferation was assessed using the SRB assay after acute exposure. Data are presented as mean ± SD of three independent replicates. Differences were considered statistically significant (*p* < 0.05).

The effect of acute exposure to PFOA, PFOS, and their mixture (PFOA and PFOS) was compared with that of the control. It was observed that MOSECs exposed to PFOS showed a 15% proliferation increase compared to the control. In the mixture group, a 20–25% increase in proliferation was observed. In contrast, PFOA-exposed cells showed a 15–20% decrease in cell proliferation (see Figure 1).

**Figure 2:**
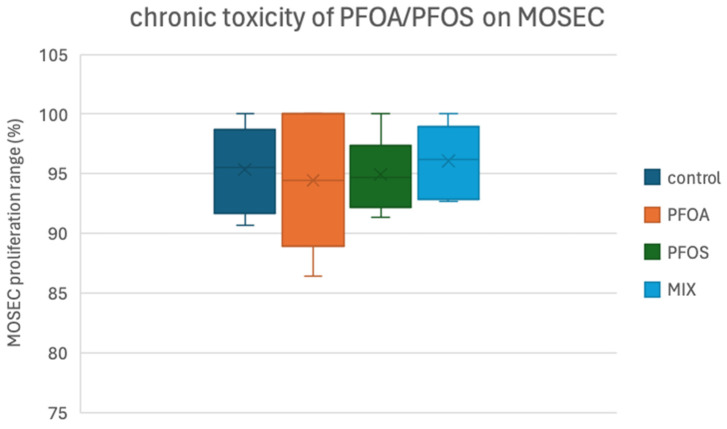
Chronic toxicity of PFOA/PFOS on MOSEC: A 28-day continuous exposure of MOSEC to PFOA, PFOS, and their mixture, % proliferation was assessed using SRB. Data are presented as amean ± SD of three independent replicates. Differences were considered statistically significant (*p* < 0.05).

In contrast to acute exposure, chronic exposure with the same concentration of PFOA, PFOS, and Mix resulted in reduced proliferation in all experimental groups. No increase in proliferation was observed in the PFOS or PFAS mixture group (see Figure 2).

Drug interactions: Effects of cabazitaxel or docetaxel on acute and chronic PFAS-exposed MOSECs.

**Figure 3:**
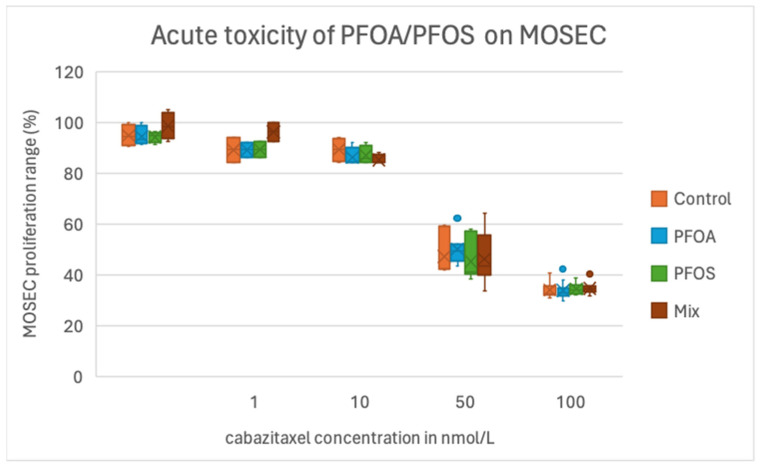
Acute toxicity of PFOA/PFOS on MOSEC treated with cabazitaxel doses of 1, 10, 50, 100 nmol/L. % proliferation was assessed using SRB. Data are presented as mean ± SD of three independent replicates. Differences were considered statistically significant (*p* < 0.05).

**Figure 4:**
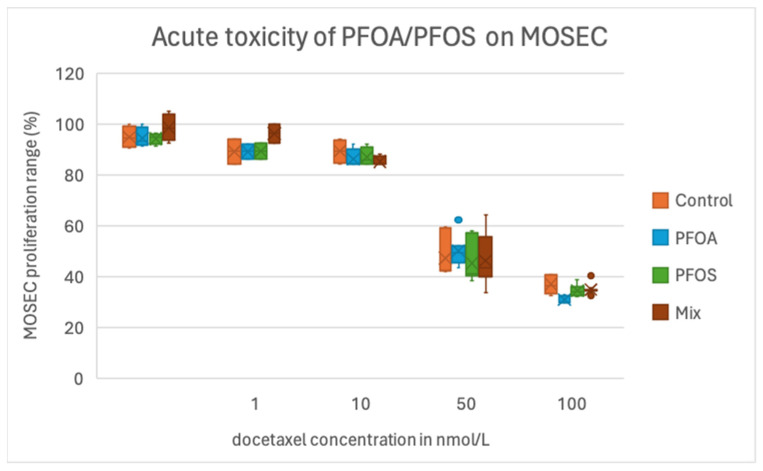
Acute toxicity of PFAS on MOSEC treated with docetaxel doses of 1, 10, 50, 100 nmol/L. % proliferation was assessed using SRB. Data are presented as mean ± SD of three independent replicates. Differences were considered statistically significant (*p* < 0.05).

The effects of cabazitaxel and docetaxel were evaluated in both acutely and chronically PFOA/PFOS-exposed MOSECs and compared with non-exposed controls. In acutely exposed cells, drug-induced cytotoxicity was observed starting from 50 nmol/L, showing a dose-dependent decrease in cell viability (see Figure 3 and Figure 4).

**Figure 5:**
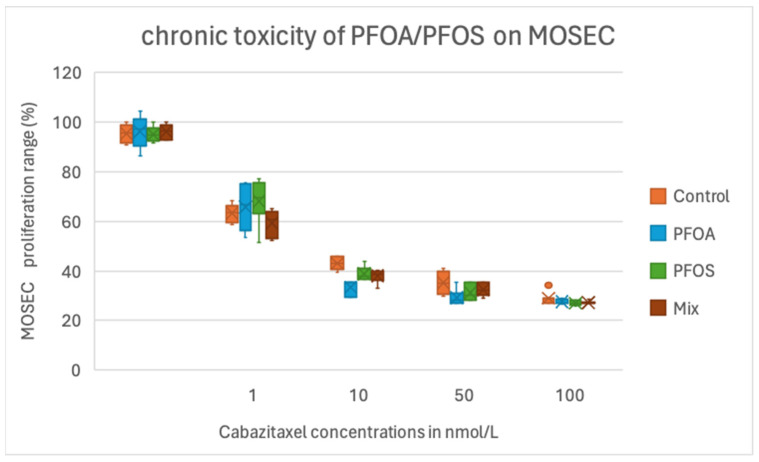
Chronic toxicity of PFAS on MOSEC treated with cabazitaxel doses of 1, 10, 50, and 100 nmol/L. % proliferation was assessed using SRB. Data are presented as mean ± SD of three independent replicates. Differences were considered statistically significant (*p* < 0.05).

**Figure 6:**
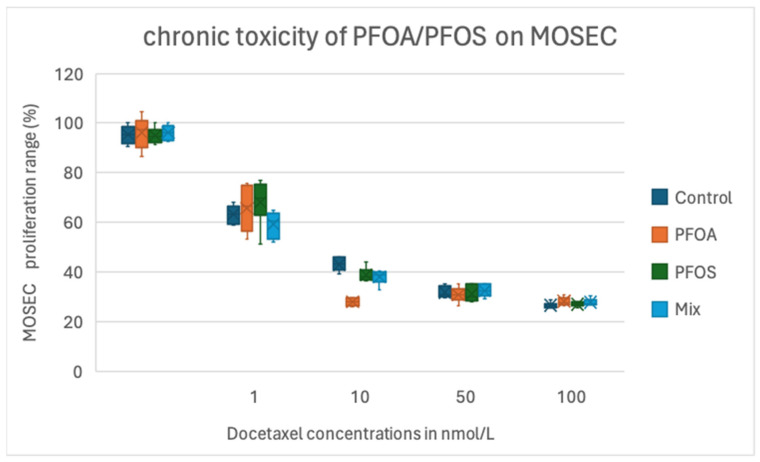
Chronic toxicity of PFAS on MOSEC treated with docetaxel doses of 1, 10, 50, and 100 nmol/L. % proliferation was assessed using SRB. Data are presented as mean ± SD of three independent replicates. Differences were considered statistically significant (*p* < 0.05).

In contrast, chronically exposed cells exhibited a cytotoxic response at lower drug concentrations (1 and 10 nmol/L) than acutely exposed cells (Figure 5 and Figure 6).

## 3. Discussion

In our study, we demonstrated that acute exposure to PFOA/PFOS significantly altered MOSEC proliferation. Specifically, PFOS-induced cell proliferation was further enhanced in the presence of PFOA, whereas PFOA alone exhibited a reduction in cell proliferation, suggesting compound-specific effects.

The proliferative potential of PFOS observed here is consistent with the reported suppression of tumour suppressor genes (*P53*, *P27*) [39]. Previous studies have suggested that PFOS exposure may activate the PI3k/Akt/RRMI-associated signalling pathways linked to neoplastic proliferation and chemoresistance [40,41,42,43,44,45].

This proposed mechanistic explanation was not directly measured in this study but rather was based on previous studies; hence, it remains speculative.

The observed pro-proliferative effect of PFOS and the differential effects of PFOA and PFOS may reflect differences in their chemical structure, protein-binding behaviour, and cellular interactions. Additionally, PFOS-induced cysteine metabolism may strengthen antioxidant defence mechanisms, thereby promoting cell survival and proliferation [46].

Acute PFOA exposure resulted in decreased cell proliferation, which is consistent with previous studies [47,48]. Although the mechanistic pathways were not directly performed, previous studies have demonstrated that PFOA exposure may contribute to oxidative stress and genotoxic effects in the reproductive system and potentially alter chromatin-associated interactions and cellular homeostasis [33]; it is also associated with increased levels of lipoperixodise and decreased antioxidant capacity [49]. However, our findings during chronic exposure differ from previous observations, where sustained proliferation was reported [32].

This discrepancy may suggest that chronic PFOA/PFOS induces a metabolic exhaustion phase, leading to a shift from proliferation to increased drug sensitivity. The contrasting responses observed following acute and chronic PFAS exposure suggest that exposure duration is an important determinant of cellular behaviour, while acute exposure may trigger a transient adaptive response that favours cell survival and proliferation. Prolonged exposure may result in cumulative cellular stress and altered homeostatic regulation. These adaptive changes may contribute to the reduced proliferation and increased cell sensitivity to drugs, as observed in chronic exposure.

This chronic effect may potentially involve reactive oxygen species (ROS) accumulation and oxidative-mediated DNA damage, which lowers the apoptosis threshold and increases chemotherapy sensitivity [50].

In contrast, acute exposure may promote rapid proliferative signalling, resulting in transient drug resistance.

Regarding drug interactions, acute PFOA/PFOS exposure reduced sensitivity to cabazitaxel or docetaxel; at low concentrations (1 and 10 nmol/L), neither drug showed a significant cytotoxic effect, whereas higher concentrations (50 and 100 nmol/L) induced dose-dependent cytotoxicity.

This acute resistance is consistent with previous findings suggesting that PFAS exposure may promote chemotherapy resistance [30]. To further evaluate the drug sensitivity, estimated IC50 values of cabazitaxel and docetaxel were calculated from dose–response data.

See Table 1 (Estimated IC50 values nmol/L of cabazitaxel and docetaxel following acute and chronic exposure to PFOA, PFOS, and their mixture).

The acute exposure group exhibited IC50 values ranging from 44 to 50 nmol/L, whereas chronic exposure groups exhibited marked lower IC50 values ranging from 5 to 8 nmol/L for both cabazitaxel and docetaxel.

These findings indicated that in chronic exposure, the proliferative effects of PFOS and the mixture were no longer observed, and drug cytotoxicity at lower doses became evident, indicating increased sensitivity. This shift may be related to adaptive cellular responses and stress-induced pathways, including ROS-mediated signalling. These findings suggest that serum PFOA/PFOS contamination may potentially modulate both cell survival and taxane sensitivity under experimental conditions. Interestingly, chronically cultured control cells also exhibited greater sensitivity to taxane than acute control; this observation may suggest that prolonged culture may also influence cellular responsiveness to taxane treatment. Nevertheless, a significant difference between acute and chronic PFAS-exposed groups was observed across all tested conditions.

### Human Implications of the Findings

Although the results were obtained using a murine ovarian epithelial cell in vitro model, the proliferation and chemotherapeutic response following human serum-inspired PFAS concentrations may have a potential impact on human health in chronic conditions, considering the bioaccumulative nature of PFAS.

While the current study was a preliminary in vitro-based study, the findings suggest that prolonged low-dose PFOA/PFOS exposure may influence cellular behaviour, warranting further investigation using a human-derived model and mechanistic approaches.

Limitations should be acknowledged. This study was conducted using a single cell line (MOSEC) and a unified concentration of PFOA/PFOS, which may not reflect the complexity of human ovarian physiology. Interaction between PFASs, serum proteins, and chemotherapeutic agents may also alter bioavailability. Although measuring actual PFAS concentrations in culture media would be beneficial, nominal concentrations may not reflect dynamic physiological exposure, where continuous distribution and elimination occur. The interaction, like Bliss or Loewe models, can be employed to determine the synergistic interaction between the compounds. Finally, while PI3K/Akt/RRM1 signalling is proposed, additional pathways likely contribute, and further mechanistic studies are required before extrapolation to human clinical scenarios.

## 4. Materials and Methods

### 4.1. Equipment and Chemicals

The following equipment was used in the experiment: 96-well clear flat-bottom polystyrene tissue-culture plates, cell culture flask (T25), multichannel electronic pipette, CO_2_ incubator (CBF 260, Binder, Tuttligen, Germany), microscope, spectrophotometer, cell counter Luna^®^ (LUNA-IITM Automated Cell Counter, Logos Biosystems, Villeneuve-d’Ascq, France), multi-well microplate reader, biosafety cabinet, analytical balance (Sartorius, Göttingen, Germany).

Chemicals used include the following: dimethyl sulfoxide DMSO (purity 99.5% molecular biology grade, Merck, Germany), Dulbecco’s Modified Eagle’s Medium (DMEM), penicillin–streptomycin(P/S), Dulbecco’s Phosphate-Buffered Saline (PBS), trypsin solution CAS number: 9002-07-7, Foetal bovine serum (FBS), Trypan blue CAS number: 72-75-1, Trichloroacetic acid TCA CAS number: 76-03-9 (purity 99.0%, Merck, Darmtsdat, Germany), Sulforhodamine B sodium salt SRB CAS number: 3520-42-1 (suitable for cell culture, Merck, Darmtsdat, Germany), Acetic acid CAS number:64-19-7, Tris base solution CAS number: 77-86-1, ionised water from Millipore^®^ deionizer (milli-Q^®^ IQ 7000, Merck, Darmtsdat, Germany).

### 4.2. Drugs and PFAS

Cabazitaxel CAS numbers 183133-96-2 and docetaxel CAS numbers 114977-28-5 used in the study were purchased from Merck (Darmstadt, Germany). Cabazitaxel and docetaxel are taxane chemotherapeutic agents used to treat cancers such as ovarian and prostate cancer, which are known to stabilise microtubules, induce G2/M cell-cycle arrest, and promote apoptotic signalling pathways [51]. Cabazitaxel is a semi-synthetic taxane agent used in docetaxel-resistant cancer, and both are effective antineoplastic agents. Stock solutions of both drugs were prepared at an initial concentration of 1 mg/mL by weighing 10 mg of each compound using an analytical balance (Quintix^®^ 35–1S, Sartorius, Göttingen, Germany) and dissolving them in DMSO as stock solutions. The working solution was obtained by serial dilution of the stock to get the following concentrations: 1, 10, 50, and 100 nmol/L, using DMEM.

PFAS: PFOA CAS number: 335-67-1 (purity 96%, Merck, Darmstadt, Germany); PFOS CAS number: 1763-23-1 (purity 97.9%, analytical standard, Merck, Darmstadt, Germany). Primary stock solutions of PFOA and PFOS were prepared using DMSO; intermediate working solutions were then generated by serial dilution in DMEM to yield final concentrations of 3 nmol/L PFOA, 5 nmol/L PFOS, and a mixture (3 nmol/L PFOA and 5 nmol/L PFOS). Vehicle controls received an equivalent concentration of DMSO, less than 0.01%, to ensure that the findings were not affected by solvents. The single desired concentrations of PFOA/PFOS and their mixture were chosen after multiple literature reviews reflecting the median human serum concentrations of PFOA and PFOS, allowing direct evaluation of how such low-dose levels can influence cell and drug response. This single dose selected represents concentrations reported in human biomonitoring studies [52,53].

### 4.3. Cell Line

The MOSEC line used in the study was obtained from Merck, Darmstadt, Germany, and is closely related to human ovarian surface epithelium, sharing several physiologically and biologically characteristic with human epithelial ovarian cancer [54]. MOSECs can form extensive tumours within the peritoneal cavity, similar to those seen in women with stage III and IV cancer, and MOSEC can also produce tumours in mice with intact immune systems. This makes MOSEC a useful in vitro model for investigating ovarian epithelial cellular response relevant to human disease [55].

### 4.4. Cell Preparation and Exposure to PFOA/PFOS

The MOSEC (Catalogue number—SCC145) was obtained from Sigma-Aldrich (Merck KGaA, Darmstadt, Germany). Cells were cultured under the manufacturer’s recommended conditions using Dulbecco’s Modified Eagle Medium (DMEM) supplemented with 10% foetal bovine serum (FBS) and 1% penicillin/streptomycin (P/S). Cell viability was assessed to be 98% before culture using the cell counter Luna^®^ (LUNA-IITM Automated Cell Counter, Logos Biosystems, Villeneuve-d’Ascq, France.

Cultures were maintained in T25 cell culture flasks (25 cm^2^) under sterile conditions in a laminar flow cabinet throughout the experimental period to minimise contamination. The cell line was verified to be free of mycoplasma and interspecies contamination by the manufacturer prior to delivery.

Acute exposure study: Cells were seeded into 96-well plates at a density of 2500 cells per well (100 µL) and incubated for 24 h to ensure proper cell adhesion. The media was then replaced with fresh media and incubated for an additional 24 h. Subsequently, cells were treated with cabazitaxel or docetaxel at concentrations of 1, 10, 50, and 100 nmol/L for a 24 h exposure period. Cell viability was assessed at the 72 h mark post-seeding for treated and non-treated cells, serving as controls. Experimental controls included a negative control (cells in culture media only) to establish baseline viability, a PFOA/PFOS control (cells treated with PFOA/PFOS without taxanes) to account for independent PFOA/PFOS impact. These drug concentrations were selected based on the pharmacologically relevant ranges established in previous in vitro studies [32,56]. This sub-cytotoxic range was chosen to specifically investigate microtubule stabilisation and G2/M phase arrest-mediated apoptosis, characterising the clinical efficacy of taxanes rather than generalised non-specific cell death.

### 4.5. Chronic Exposure

The purpose of the Chronic Enrichment Phase (days 1–28) was to investigate long-term background exposure, and cells were maintained in continuous culture for 28 days under the same conditions: standard culture medium (Control), PFOA-supplemented medium, PFOS-supplemented medium, and a binary mixture (PFOA + PFOS). To ensure a stable chemical equilibrium and mitigate confounding factors such as hydrophobic adsorption to plastic surfaces or potential chemical degradation, all media were prepared freshly on the morning of use. Cells were cultured in T25 flasks and sub-cultured every 72 h at a 1:4 (25%) split ratio.

This frequent passaging and transfer to a new culture flask ensured that the observed outcomes reflected the impact of stable, physiologically relevant concentrations without the interference of nutrient depletion or accumulated metabolic waste while maintaining the steady-state equilibrium between the chemicals and serum proteins (FBS) [57], thereby mimicking the stable, physiologically relevant background exposure found in human serum. The 28-day duration was chosen to allow for cellular adaptation, leveraging the known thermal and chemical stability of PFOA/PFOS [8,9].

During the Acute Drug challenge phase (days 29–30) following the enrichment period, cells from all four chronic groups were harvested and seeded into 96-well plates at a density of 2500 cells per well (100 µL/well). The cells were incubated for 24 h to ensure proper adhesion and stabilisation. Subsequently, cells were subjected to an acute challenge with cabazitaxel or docetaxel at concentrations of 1, 10, 50, and 100 nmol/L. Drug concentrations were prepared fresh before treatment to maintain drug stability and experimental consistency. Each chronic group was compared against its own intra-group control (PFOA/PFOS-exposed cells without drug treatment) to isolate taxane-specific cytotoxicity.

### 4.6. Sulforhodamine B (SRB) Protocol Assay

The SRB assay and 24 h endpoint were selected to measure the integrated low-dose PFAS impact on total biomass [58]. This method evaluates long-term population dynamics at PFOA/PFOS levels clinically relevant to humans [59], offering superior reproducibility over metabolic assays (e.g., 3-(4,5-dimethylthiazol-2-yl)-2,5-diphenyltetrazolium bromide (MTT assay), which PFOA/PFOS can confound. Upon completion of cell culture with the desired drug concentration and duration, the analysis was done as follows.

Cell fixation and staining: Each well was emptied of the old medium, and TCA was added directly into each well, and incubated at 4 °C for 1 h. The plates were washed four times by submerging them in a tub with slow-running ionised water. It was ensured that the water was slow-running to avoid cell monolayer detachment, and plates were allowed to air-dry at room temperature for proper fixation. The fixed cells were stained with 100 μL of 0.04% SRB solution, which was added to each well of the dried plates using a multi-pipette. The SRB dyes bind to the cellular proteins under mild acidic conditions, resulting in staining that is directly proportional to the number of viable cells within the culture, and the excess dye was removed with 1% acetic acid. The bound proteins were solubilized by adding 100 μL of 10 mM Tris base solution (pH 10.5) to each well.

The solution absorbance was measured spectrophotometrically at a wavelength of 570 nm using a microplate reader. The absorbance results correlate to the number of viable cells and were used to calculate the % proliferation and compare it to the control MOSEC, which was neither exposed to PFOA/PFOS nor drugs.

### 4.7. Statistical Analysis

Experiments were conducted in three entirely independent runs (N = 3) to ensure reproducibility; they were performed as separate temporal blocks under identical conditions using the same cell passage. Within each independent experiment, six technical replicates (wells) were included per treatment group. To eliminate intra-experimental variance and avoid pseudoreplication, the six technical replicates were averaged within each experiment. The resulting three independent experimental means (N = 3) were used as the statistical units for all subsequent analyses, thereby controlling for the independent experiment factor. Data are expressed as mean ± standard deviation (SD) of these independent blocks. Statistical differences among the treatment groups (Control, PFOA, PFOS, and Mix) were evaluated via separate one-way analyses of variance (ANOVA) for each designated concentration (1, 10, 50, and 100 nmol/L) and exposure duration (acute and chronic). Tukey’s Honest Significant Difference (HSD) post hoc test was applied for pairwise comparisons (PFOA vs. Control, PFOS vs. Control, Mix vs. Control, Mix vs. PFOS, and Mix vs. PFOA). Statistical analyses were performed using IBM SPSS Statistics (Version 29), with significance defined as *p* < 0.05. For cabazitaxel and docetaxel, the half-maximal inhibitory concentration (IC_50_) values were determined from the dose–response data relative to the untreated vehicle control (0 μM). Concentrations were log-transformed, and IC_50_ values were calculated by fitting the linear portion of the log-dose response data to a linear regression model using Microsoft Excel 2024 (Microsoft Corporation, Redmond, WA, USA).

## 5. Conclusions and Future Direction

This study demonstrated that acute and chronic PFOA/PFOS exposure exert differential time-dependent effects on ovarian cell viability. The enhanced proliferative effect observed in the PFOA-PFOS mixture compared with the individually exposed cell line suggests the potential interaction between the compounds. It shows that environmental contaminants may enhance proliferative capacity, which may potentially occur through suppression of tumour suppressor genes (*P53*, *P27*) and activation of PI3K/Akt/RRM1 signalling. In contrast, chronic exposure appears to trigger adaptive mechanisms, resulting in reduced proliferation and increased drug sensitivity.

Importantly, acute PFOA/PFOS exposure decreased sensitivity to cabazitaxel and docetaxel, whereas chronic exposure increased cytotoxic response, indicating a time-dependent shift in chemotherapy effectiveness.

Future studies should focus on subclinical PFAS concentrations that trigger this toxicological transition, particularly during sensitive developmental windows. Validation using primary human ovarian cells, 3D organoid models, and in vivo models is required, as these findings may have implications for chemotherapy response and environmental exposure to PFOA/PFOS.

## Figures and Tables

**Table 1:** Estimated IC50 values (nmol/L) of cabazitaxel and docetaxel following acute and chronic exposure to PFOA, PFOS, and their mixture.

Treatment Group	Acute Cabazitaxel IC50 (nmol/L)	Acute Docetaxel IC50 (nmol/L)	Chronic Cabazitaxel IC50 (nmol/L)	Chronic Docetaxel IC50 (nmol/L)
Control	47	47	8	8
PFOA	50	49	5	5
PFOS	45	44	6	7
Mix	45	45	7	7

## Data Availability

The raw data supporting the findings of this study (SRB assay measurements and cell proliferation calculations) are available from the corresponding author upon reasonable request.
